# Identification of an Immune-Related Risk Signature Correlates With Immunophenotype and Predicts Anti-PD-L1 Efficacy of Urothelial Cancer

**DOI:** 10.3389/fcell.2021.646982

**Published:** 2021-03-18

**Authors:** Pengju Li, Shihui Hao, Yongkang Ye, Jinhuan Wei, Yiming Tang, Lei Tan, Zhuangyao Liao, Mingxiao Zhang, Jiaying Li, Chengpeng Gui, Jiefei Xiao, Yong Huang, Xu Chen, Jiazheng Cao, Junhang Luo, Wei Chen

**Affiliations:** ^1^Department of Urology, First Affiliated Hospital of Sun Yat-sen University, Guangzhou, China; ^2^Institute of Precision Medicine, The First Affiliated Hospital, Sun Yat-sen University, Guangzhou, China; ^3^State Key Laboratory of Oncology in South China, Collaborative Innovation Center for Cancer Medicine, Sun Yat-sen University Cancer Center, Guangzhou, China; ^4^Department of Urology, Dongguan People’s Hospital, Affiliated to Southern Medical University, Dongguan, China; ^5^Department of Extracorporeal Circulation, First Affiliated Hospital of Sun Yat-sen University, Guangzhou, China; ^6^Department of Urology, Jiangmen Central Hospital, Affiliated Jiangmen Hospital of Sun Yet-sen University, Jiangmen, China

**Keywords:** immune-related risk signature, immunity gene, immune checkpoint inhibitor, urothelial cancer, tumor microenvironment

## Abstract

Immune checkpoint inhibitor (ICI) treatment has been used to treat advanced urothelial cancer. Molecular markers might improve risk stratification and prediction of ICI benefit for urothelial cancer patients. We analyzed 406 cases of bladder urothelial cancer from The Cancer Genome Atlas (TCGA) data set and identified 161 messenger RNAs (mRNAs) as differentially expressed immunity genes (DEIGs). Using the LASSO Cox regression model, an eight-mRNA-based risk signature was built. We validated the prognostic and predictive accuracy of this immune-related risk signature in 348 metastatic urothelial cancer (mUC) samples treated with anti-PD-L1 (atezolizumab) from IMvigor210. We built an immune-related risk signature based on the eight mRNAs: ANXA1, IL22, IL9R, KLRK1, LRP1, NRG3, SEMA6D, and STAP2. The eight-mRNA-based risk signature successfully categorizes patients into high-risk and low-risk groups. Overall survival was significantly different between these groups, regardless if the initial TCGA training set, the internal TCGA testing set, all TCGA set, or the ICI treatment set. The hazard ratio (HR) of the high-risk group to the low-risk group was 3.65 (*p* < 0.0001), 2.56 (*p* < 0.0001), 3.36 (*p* < 0.0001), and 2.42 (*p* = 0.0009). The risk signature was an independent prognostic factor for prediction survival. Moreover, the risk signature was related to immunity characteristics. In different tumor mutational burden (TMB) subgroups, it successfully categorizes patients into high-risk and low-risk groups, with significant differences of clinical outcome. Our eight-mRNA-based risk signature is a stable biomarker for urothelial cancer and might be able to predict which patients benefit from ICI treatment. It might play a role in precision individualized immunotherapy.

## Introduction

Bladder cancer is a common tumor of the urinary system; 90% of the pathological types are urothelial cancer (UC). For bladder cancer patients with local progression or distant metastasis, cisplatin combined with gemcitabine is the first choice. However, the effect is not satisfactory. The median survival time of patients is only 15 months, and the 5 years survival rate is difficult to reach 15% (Griffiths et al., 2011). In recent years, with the rapid development of tumor immune checkpoint inhibitors (ICIs), especially the rapid development of programmed cell death molecule 1 (PD-1)/programmed cell death molecule ligand 1 (PD-L1) inhibitors, the treatment of bladder cancer patients has brought new options. In patients with metastatic UC (mUC) who cannot receive cisplatin chemotherapy and PD-L1 positive, ICIs can already be used as a first-line treatment (Nadal and Bellmunt, 2019).

Although it is proved the efficacy of PD-1/PD-L1 inhibitors is better than that of traditional platinum-based chemotherapy (Bellmunt et al., 2017; Sidaway, 2017), studies have confirmed that only about 20% of solid tumor patients can benefit from the treatment (Braun et al., 2016). Therefore, it is becoming more and more important to identify and verify biomarkers that can accurately predict ICI treatment efficacy. There have been some clinical studies exploring the corresponding biomarkers, such as PD-L1 expression (Powles et al., 2014), CD8^+^ T cell (Ghatalia and Plimack, 2019), tumor mutational burden (TMB) (Yarchoan et al., 2017), and microsatellite instability (MSI) (Dudley et al., 2016). However, these biomarkers have shortcomings in clinical application. In addition, multi-factor joint prediction may be able to provide better prediction results (Mazzaschi et al., 2020).

In this study, we analyzed the messenger RNA (mRNA) transcriptome data of 406 bladder cancer patients from The Cancer Genome Atlas (TCGA) data combined with immune-related genes to establish an immune-related risk signature, and we verified the 348 mUC patients receiving anti-PD-L1 therapy from IMvigor210 study. We emphasized the strong predictive power of the risk score in selecting patients with good response to atezolizumab and verified its role in ICI treatment.

## Materials and Methods

### Clinical Cohorts and Data Sets

The gene expression sequence matrix and clinical characteristics of 406 bladder cancer patients can be downloaded from TCGA data set^1^. The immune gene sets come from the ImmPort^2^ and InnateDB^3^ data sets (Breuer et al., 2013; Bhattacharya et al., 2014). Clinical information and gene transcription information of 348 patients with mUC who received ICI treatment are downloaded from^4^ (Mariathasan et al., 2018). The infiltration of 22 immune cells was downloaded from the TIMER database^5^ and Dongqiang Zeng’s research (Li T. et al., 2020; Zeng et al., 2020).

### Bioinformatic Analysis

R package DESeq2 was used for gene expression differential analysis, and R package clusterProfiler for Gene Ontology/Kyoto Encyclopedia of Genes and Genomes/Gene Set Enrichment Analysis (GO/KEGG/GSEA) function enrichment analysis and visualization (Subramanian et al., 2005; Love et al., 2014). The ggstatsplot package was used to evaluate the relationship between risk score, TMB, and immunophenotype.

### Establishment and Evaluation of Risk Prediction Model

We randomly divide the samples in TCGA cohort into training/validation (3:1) groups to identify and evaluate predictors. The “glmnet” R package was used for LASSO analysis, and 14 immune-related genes were identified (Ternes et al., 2016). Then we conducted multiple Cox regression analysis to establish an eight-mRNA-based risk prediction model. Use the formula to generate the risk score for each patient: risk score = EXP 1 ^∗^ β 1 EXP2 ^∗^ β 2 … EXPÑ ^∗^ β Ñ, where “EXP” represents the expression level of key genes and β is the corresponding regression coefficient. The “timeROC” package was used to establish the receiver operating characteristic (ROC) curve and verify the area value under it (AUC). Draw a Kaplan–Meier curve to show the association of risk scores and potential prognostic genes with patient survival.

### Statistical Analysis

The Kaplan–Meier method was used to analyze the correlation between relate risk factors with patient survival. Statistical tests were performed using R software, version 3.6. 2 (R Foundation for Statistical Computing; Vienna, Austria). Values of *p* < 0.05 were considered statistically significant.

## Results

### Describe Immune-Related Gene Features and Construct Immune-Related Prediction Models

**FIGURE 1:**
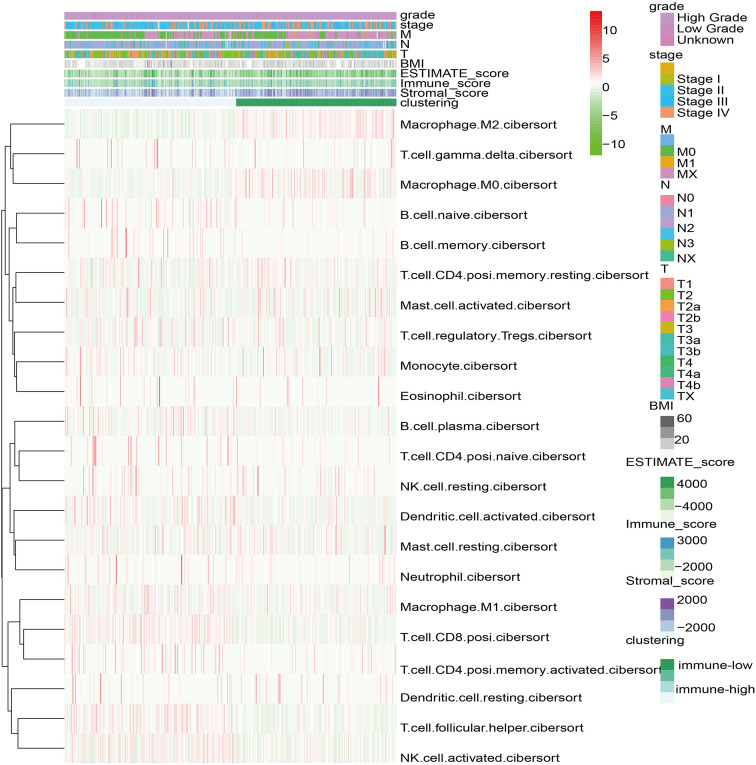
Heatmap of 22 immune cell types based on immune microenvironment clustering. Missing clinical data are shown as blank on the top of the heatmap.

Based on the Cibersort algorithm, the infiltration levels of 22 immune cells in 406 bladder cancer patients in TCGA data set were evaluated. The fuzzy clustering algorithm divided the samples into two categories: 210 samples were clustered into cluster 1 and 196 samples were clustered into cluster 2 (Figure 1). Cluster 1 had high-level immune characteristics (high-immunity group), and cluster 2 had low-level immune characteristics (low-immunity group). Missing clinical data are shown as blank on the top of the heatmap.

**FIGURE 2:**
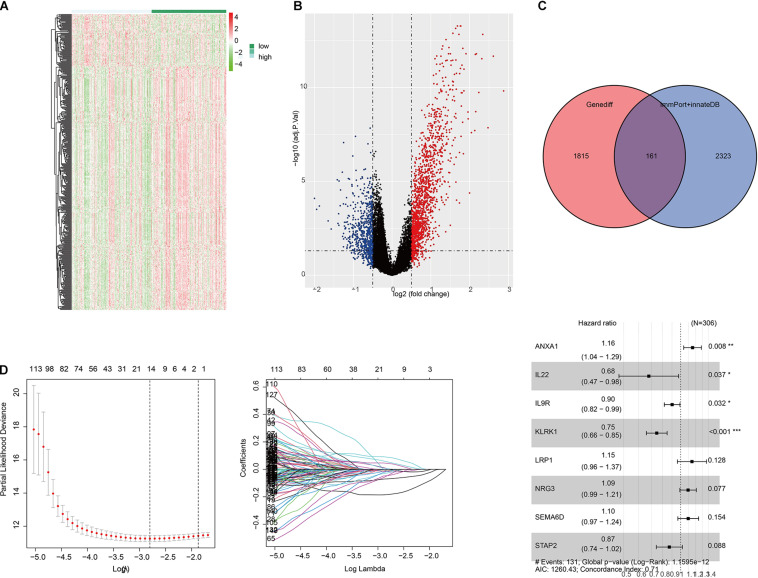
Selection of differentially expressed immunity genes (DEIGs) and establishment of immune-related risk models. **(A)** Heatmap of all differentially expressed genes (DEGs) in the immunity low group and immunity high group. **(B)** Volcano plot of all DEGs showing the log 2 fold change and *q* value of each DEG. **(C)** Venn diagram of DEGs showing immune-related gene set obtained from ImmPort and InnateDB. **(D)** LASSO coefficient distribution map, prognostic biomarker selection characteristics, and forest plot based on multivariable Cox proportional hazards regression. *p < 0.05, **p < 0.01, ***p < 0.001.

We were trying to study immune-related genes stratified by immune phenotype and their prognostic potential and to establish a good immunological prediction model, which can make more accurate individual risk stratification and prognosis prediction for UC patients. After preliminary screening through single-factor regression analysis and difference analysis based on immunity clustering, a total of 1,976 genes were identified as differentially expressed genes (DEGs). All DEG expression levels are shown in Figure 2A, and the log2 and *p*-values of all DEGs are shown in Figure 2B. Subsequently, 161 genes were identified as differentially expressed immunity genes (DEIGs) based on ImmPort and InnateDB databases (Figure 2C). In order to further explore the prognostic significance of DEIGs, 161 important genes were used for multiple LASSO regression and multiple Cox regression analysis (Figure 2D), and eight key DEIGs were identified. Distribution of each DEIG in TCGA set and ICI treatment set is shown in Supplementary Figure 1. The Kaplan–Meier curve of each DEIG in TCGA set is shown in Supplementary Figure 2 [ANXA1: *p* < 0.0001, hazard ratio (HR) = 2.02; IL22: *p* < 0.0001, HR = 0.50; IL9R: *p* < 0.0001, HR = 0.71; KLRK1: *p* < 0.0001, HR = 0.54; LRP1: *p* = 0.0051, HR = 1.96; NRG3: *p* = 0.00065, HR = 1.40; SEMA6D: *p* = 0.01, HR = 1.65; STAP2: *p* = 0.00029, HR = 0.68]. Finally, according to the relative coefficient in the multiple regression analysis, the risk score was calculated according to the following formula: (0.144493498 ^∗^ ANXA1) + (−0.387235675 ^∗^ IL22) + (−0.103863619 ^∗^ IL9R) + (−0.291034924 ^∗^ KLRK1) + (0.137267967 ^∗^ LRP1) + (0.090089369 ^∗^ NRG3) + (0.091881101 ^∗^ SEMA6D) + (−0.138073124 ^∗^ STAP2) (Table 1).

**TABLE 1:** Characteristics of differentially expressed immunity genes (DEIGs) in the risk signature.

	coef	HR	HR.95L	*p*-value
ANXA1	0.1444935	1.16	1.04–1.29	0.0085
IL22	–0.3872357	0.68	0.47–0.98	0.0365
IL9R	–0.1038636	0.9	0.82–0.99	0.0325
KLRK1	–0.2910349	0.75	0.66–0.85	<0.0001
LRP1	0.13726797	1.15	0.96–1.37	0.128
NRG3	0.09008937	1.09	0.99–1.21	0.0771
SEMA6D	0.0918811	1.1	0.97–1.24	0.1539
STAP2	–0.1380731	0.87	0.74–1.02	0.0878

### Survival Analysis, Prognostic Value, and Immune Infiltration Verification of the Risk Signature

**FIGURE 3:**
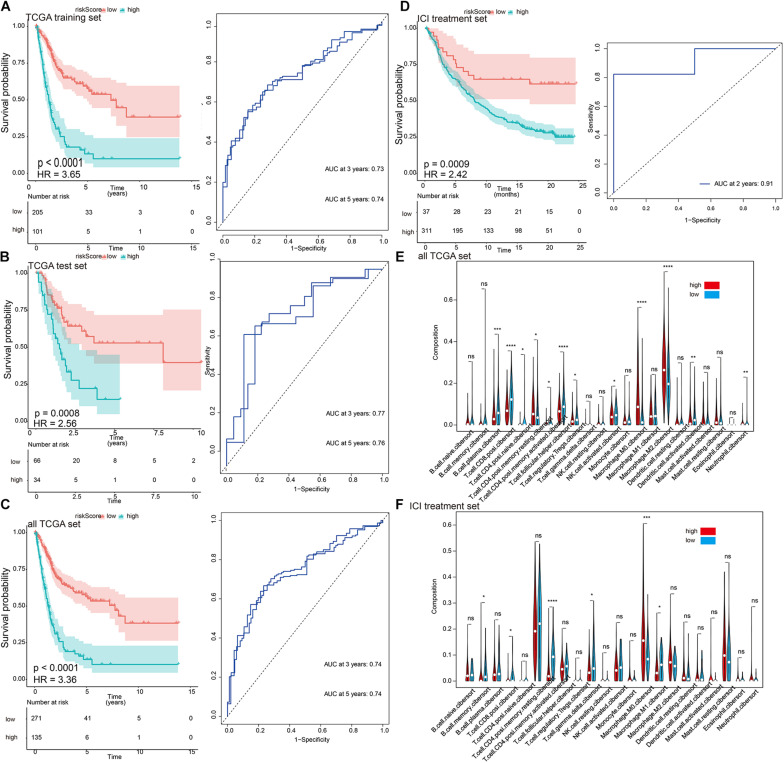
The characterization of the training and validation cohorts highlights that risk scores are potential biomarkers. **(A–D)** Kaplan–Meier survival analysis and time-dependent receiver operating characteristic (ROC) curve of the risk signature. The risk score derived from the constructed model is significantly correlated with overall survival. **(E,F)** The infiltration trends of 22 immune cells are consistent in the two data sets. *p < 0.05, **p < 0.01, ***p < 0.001, ****p < 0.0001.

The prognostic value of eight DEIG signatures was further evaluated in three verification sets (TCGA test set, all TCGA set, and independent ICI treatment set). We calculated the risk score of each patient using the same formula, and we divided them into high-risk and low-risk groups by 1.54 as a cutoff. Consistent with the results of TCGA training set, the prognosis of high-risk patients in the three validation sets was worse than that of patients in the low-risk group (Figures 3A–D, left; TCGA training set: *p* < 0.0001, HR = 3.65; TCGA test set: *p* < 0.0001, HR = 2.56; all TCGA set: *p* < 0.0001, HR = 3.36; ICI treatment set: *p* = 0.0009, HR = 2.42). The results of the time-dependent ROC curve analysis verified the predictive value of the established risk model (Figures 3A–D, right), suggesting that the prognosis prediction for 3–5 years was more robust. The univariate and multivariate Cox analyses of TCGA set showed that the risk signature can be used as an independent prognostic factor (Supplementary Table 1). We examined the correlation between risk signature and the bladder cancer immune microenvironment. Both TCGA and ICI data sets showed a relatively consistent trend of infiltration. In terms of immune cell infiltration, such as T.cell.CD8.positive, T.cell.CD4.activated, and Macrophage.M0, the infiltration trend was the same in the two data sets. The differences were statistically significant (Figures 3E,F).

### Verification and Comparison of the Correlation Between the Risk Signature and Immune Checkpoint Inhibitor Treatment Efficacy

**FIGURE 4:**
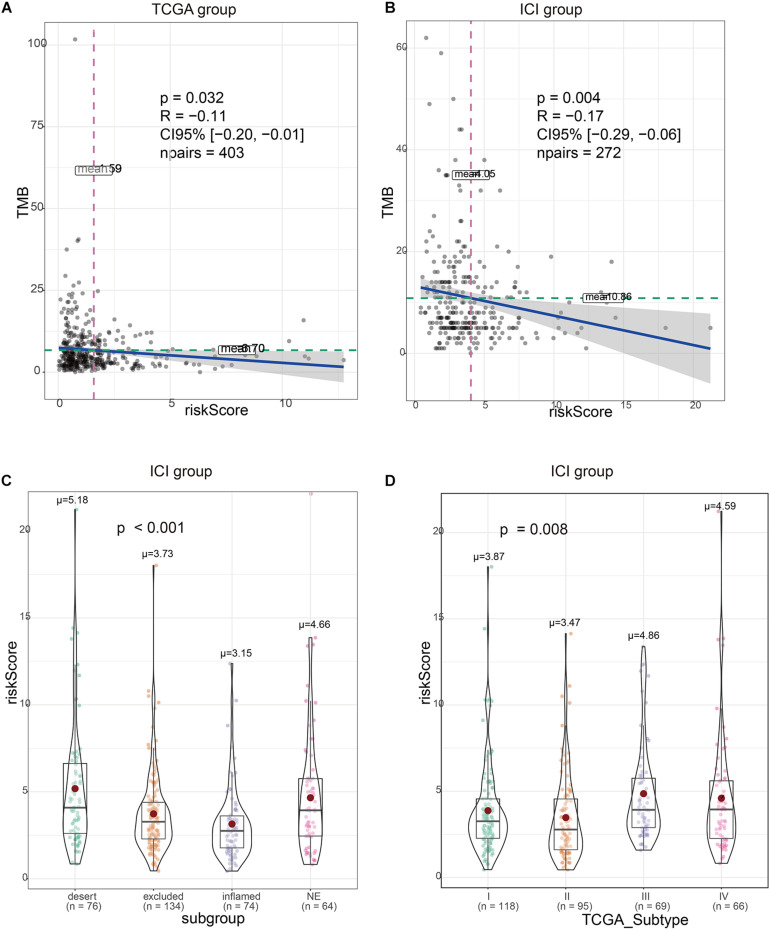
Validation of the correlation between the risk signature and immunity characteristics. **(A)** Risk scores are correlated with tumor mutational burden (TMB) in The Cancer Genome Atlas (TCGA) set. **(B)** Risk scores are correlated with TMB in immune checkpoint inhibitor (ICI) treatment set. **(C)** Risk scores are correlated with immunity subtype in ICI treatment set. **(D)** Risk scores are correlated with TCGA subtype in ICI treatment set.

There was a significant correlation between the risk signature and TMB, regardless if in TCGA or ICI treatment set (Figures 4A,B). At the same time, the immune subtypes classified according to CD8 cell infiltration (desert, excluded, and inflamed) were also obviously related to the risk signature (Figure 4C). TCGA type II subgroup had the lowest risk score (Figure 4D). This is consistent with the previous results (Mariathasan et al., 2018).

**FIGURE 5:**
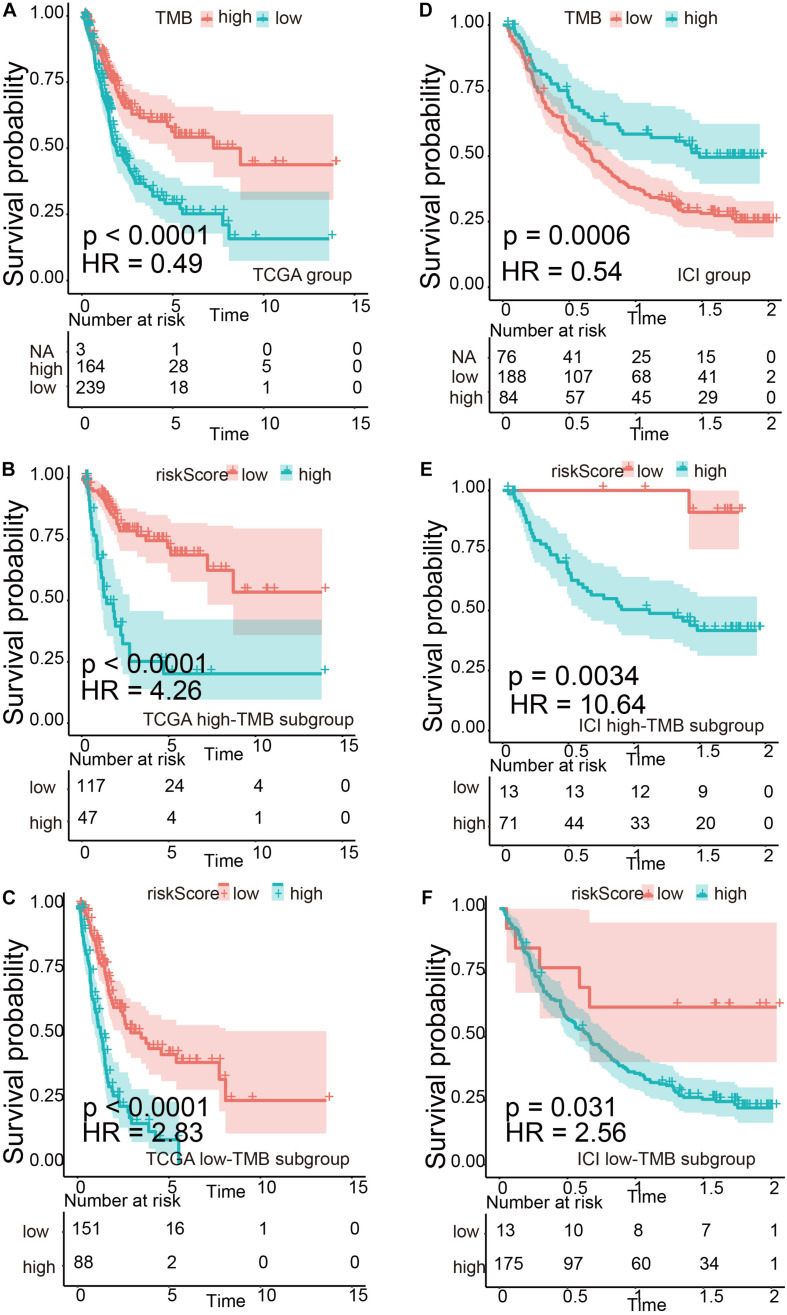
Verification of the risk signature to be used as a stable predictor. **(A–C)** Kaplan–Meier survival analysis based on tumor mutational burden (TMB) levels and TMB subgroups in The Cancer Genome Atlas (TCGA) data set. **(D–F)** Kaplan–Meier survival analysis based on TMB levels and TMB subgroups in the immune checkpoint inhibitor (ICI) treatment set.

We divided TMB into high-risk and low-risk groups and then subdivided them into subgroups based on the risk scores level (TCGA set: *p* < 0.0001, HR = 0.49; ICI treatment set: *p* = 0.0006, HR = 0.54). The results suggested that even in the TMB subgroup, the risk signature still remained its prognostic ability, regardless if in TCGA or ICI treatment set (Figure 5, TCGA high-TMB group: *p* < 0.0001, HR = 4.26; TCGA low-TMB group: *p* < 0.0001, HR = 2.83; ICI treatment high-TMB group: *p* = 0.0034, HR = 10.64; ICI treatment low-TMB group: *p* = 0.031, HR = 2.56). Multivariate risk regression also confirmed this result (Table 2).

**TABLE 2:** Multivariate Cox regression of risk scores and tumor mutational burden (TMB) in immune checkpoint inhibitor (ICI) treatment set.

	HR	95%CI	*p*-value
Risk score	4.83	2.14−10.93	0.0002
TMB	0.96	0.94−0.99	0.0012

### Gene Ontology/Kyoto Encyclopedia of Genes and Genomes/Gene Set Enrichment Analysis

**FIGURE 6:**
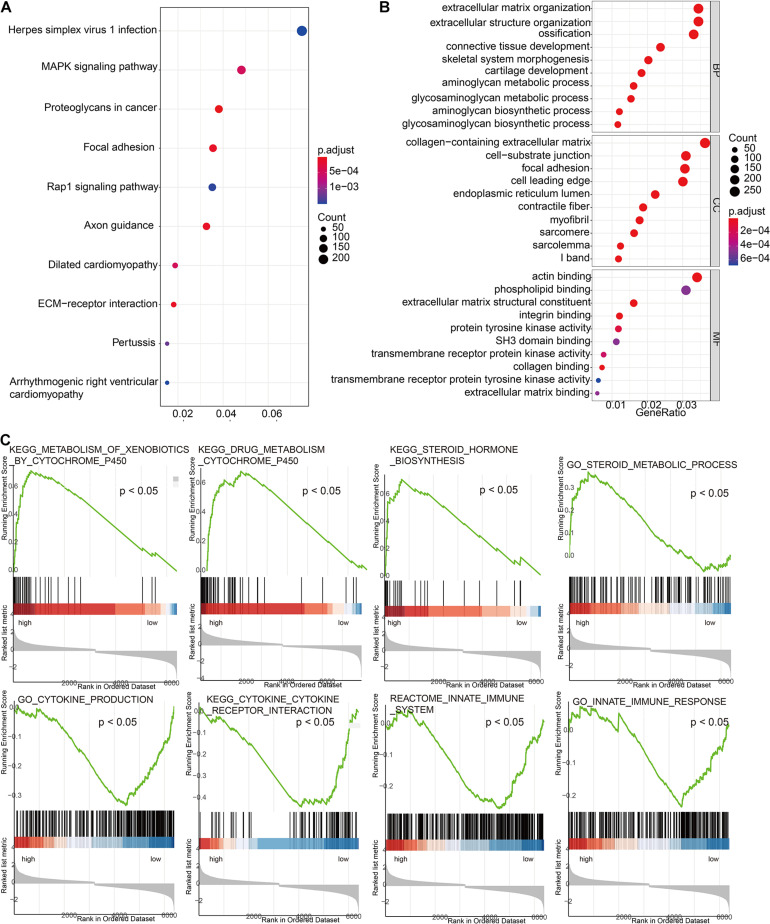
Exploration of the molecular mechanisms related to risk scores. **(A)** Gene Ontology enrichment analysis based on risk scores. **(B)** Kyoto Encyclopedia of Genes and Genomes (KEGG) enrichment analysis based on risk scores. **(C)** Gene Set Enrichment Analysis (GSEA) based on risk scores.

In order to further explore the molecular mechanisms related to risk scores, we divided TCGA cohort patients into high-risk and low-risk groups. The results of GO and KEGG suggested that the risk signature was related to the extracellular matrix and energy metabolism changes in the tumor microenvironment (Figures 6A,B). GSEA results suggested that the high-risk group is positively correlated with steroid metabolism and YP450 metabolism, while pathways such as cytokine interaction and immune response are positively correlated with low-scoring risks (Figure 6C).

## Discussion

As a new treatment method, ICI has initially proven efficacy and safety in the treatment of UC. Unfortunately, not all patients with cancer respond to ICI treatment. In previous studies, the use of risk signature derived from the gene transcriptome to monitor the immune status of tumors and guide individualized treatment has proven to be meaningful (Li et al., 2017; Cristescu et al., 2018; Wang et al., 2019). Therefore, the development of meaningful genetic markers to monitor the immune status of patients not only can monitor the prognosis of patients but also can screen out potential ICI responding patients, avoiding the waste of medical resources and overtreatment. In this study, we validated immune-related gene risk model based on eight DEIGs, which proved to be a reliable indicator of favorable ICI efficacy and can identify bladder cancer patients with poor prognosis. Our results showed that the prognosis is worse if the risk score is higher. At the same time, the time-dependent ROC curve results suggested that the 3–5 years’ prognosis prediction for UC patients was more robust. In terms of tumor immune cell infiltration, whether in TCGA group or the ICI treatment group, the differences in T.cell.CD8.positive, T.cell.CD4.activated, and Macrophage.M0 were statistically significant. Patients in the high-risk group had significantly lower representation of T.cell.CD8.positive and T.cell.CD4.activated and significantly higher abundance of Macrophage.M0. This is also consistent with the results of other studies (Li W. et al., 2020; Lin et al., 2020).

PD-L1 is currently the most mature and in-depth biomarker, but the results obtained in different ICI studies are not consistent. It may be due to the different monitoring methods and positive standards between different platforms, and the evaluation is subjective. At present, due to technical requirements, it is difficult to apply TMB to routine clinical practice (Fenizia et al., 2018). In addition, TMB has not yet proven its predictive or prognostic value for overall survival (Addeo et al., 2019). According to the immune microenvironment, most solid tumors can be divided into three different immunological phenotypes: immune inflamed, immune excluded, or immune desert (Chen and Mellman, 2013; Hegde et al., 2016). Studies have shown that immune inflamed subtypes have the best response to ICI treatments (such as anti-PD-1 and anti-CTLA-4) (Mariathasan et al., 2018; Galon and Bruni, 2019). The immune-related risk signature we established was significantly correlated with TMB and immunophenotype. A lower risk score means a higher TMB, a better response, and a better prognosis. At last, the K-M curve of TMB subtype showed that the risk signature was able to be used as a stable predictor. In order to further clarify the mechanism of immune risk score, we subsequently used TCGA data set to conduct GO, KEGG, and GSEA. The results of GO and KEGG show that the risk score is related to the energy metabolism and synthesis of the tumor microenvironment, and the formation and activation of extracellular matrix. In the analysis of GSEA results, we can see that the synthesis and metabolism of steroids are positively correlated with high-scoring risks, while pathways such as cytokine interaction and immune response are positively correlated with low-scoring risks. This is consistent with the results of other studies. Zeng et al. (2020) found that the defect of M1 macrophage function is related to poor prognosis of UC immunotherapy, and it is also positively related to steroid synthesis and metabolism.

Among the eight DEIGs, there are few studies in bladder cancer, but some of their interactions with immunity have been explored and verified in other researches. ANXA1 can enhance the function of regulatory T cells (Tregs) and reduce the survival rate of patients with breast cancer (Bai et al., 2020). IL-22 producing T cells in colorectal cancer enhance T cell function by recruiting neutrophils, thereby enhancing immune response (Tosti et al., 2020). Th9 cells promote the expansion of CD8^+^ T cells in an IL-9R-dependent manner in colorectal cancer (Wang et al., 2020). CIK cells can target lung cancer cells expressing NKG2D/KLRK1 ligand, and the killing effect can be partially blocked by NKG2D/KLRK1 ligand inhibitors (Yin et al., 2017). The correlation between LRP1 mRNA expression and patient survival was observed in bladder urothelial carcinoma. At the same time, the LRP1 protein can regulate the immune function by regulating the movement and adhesion of T cells (Gonias et al., 2017; Panezai et al., 2017). STAP2 maintains the cytotoxicity of functional memory CD8^+^ T cells by controlling cytokine signaling inhibitor 3 (Muraoka et al., 2017). SEMA6D act as a modulator in the late stage of the primary immune response (O’Connor et al., 2008).

Although the risk signature based on eight DEIGs embodies a powerful predictive function in selecting patients with good response to atezolizumab, its accuracy and effectiveness should be further verified in a prospective cohort study receiving immunotherapy. In addition, the molecular mechanism of the protein encoded by DEIGs in UC still needs to be explored *in vitro* and *in vivo*.

The risk signature is a stable biomarker that can be used to predict immunotherapy efficacy and immunophenotype determination, and it can be used as a supplement to TMB.

## Data Availability Statement

Publicly available datasets were analyzed in this study. This data can be found here: The gene expression sequence matrix and clinical characteristics of 406 bladder cancer patients was downloaded from the TCGA data set (https://portal.gdc.cancer.gov). The immune gene sets were downloaded from the Immport (https://s3.immport.org/release/genelists/GeneList.txt?download=true) and InnateDB (https://www.innatedb.com/download/innatedb_curated_genes.xls) data sets. Clinical information and gene transcription information of 348 patients with mUC who received ICI treatment was downloaded from http://research- pub.gene.com/IMvigor210CoreBiologies. The infiltration of 22 immune cells was downloaded from the TIMER database (http:// timer.cistrome.org/infiltration_estimation_for_tcga.csv.gz) and Dongqiang Zeng’s research (https://www.thno.org/v10/p7002/thnov10p7002s2.xlsx).

## Author Contributions

JLu and WC designed the study. PL, SH, YY, YT, LT, CG, and JW obtained and assembled data. PL, SH, JLi, JW, YH, ZL, and JC analyzed and interpreted the data. PL, JX, MZ, XC, and WC wrote the report. All authors approved the final version. JC, JLu, and WC are the guarantors.

## Conflict of Interest

The authors declare that the research was conducted in the absence of any commercial or financial relationships that could be construed as a potential conflict of interest.

## Footnotes

1 https://portal.gdc.cancer.gov

2 https://www.immport.org

3 http://www.innatedb.com

4 http://research-pub.gene.com/IMvigor210CoreBiologies

5 http://timer.cistrome.org

## Supplementary Material

Supplementary Figure 1 Distribution of each DEIG in TCGA set and ICI treatment set.

Supplementary Figure 2 Kaplan–Meier curve of each DEIG in TCGA set.

Supplementary Table 1 Univariate and multivariate Cox regression analysis of clinical pathologic features in TCGA set.
