# Supplementary figures and images for: Identification of an Immune-Related Risk Signature Correlates With Immunophenotype and Predicts Anti-PD-L1 Efficacy of Urothelial Cancer
Source: Front Cell Dev Biol. 2021 Mar 18;9:646982. doi: 10.3389/fcell.2021.646982 (PMC8012532; doi:10.3389/fcell.2021.646982)

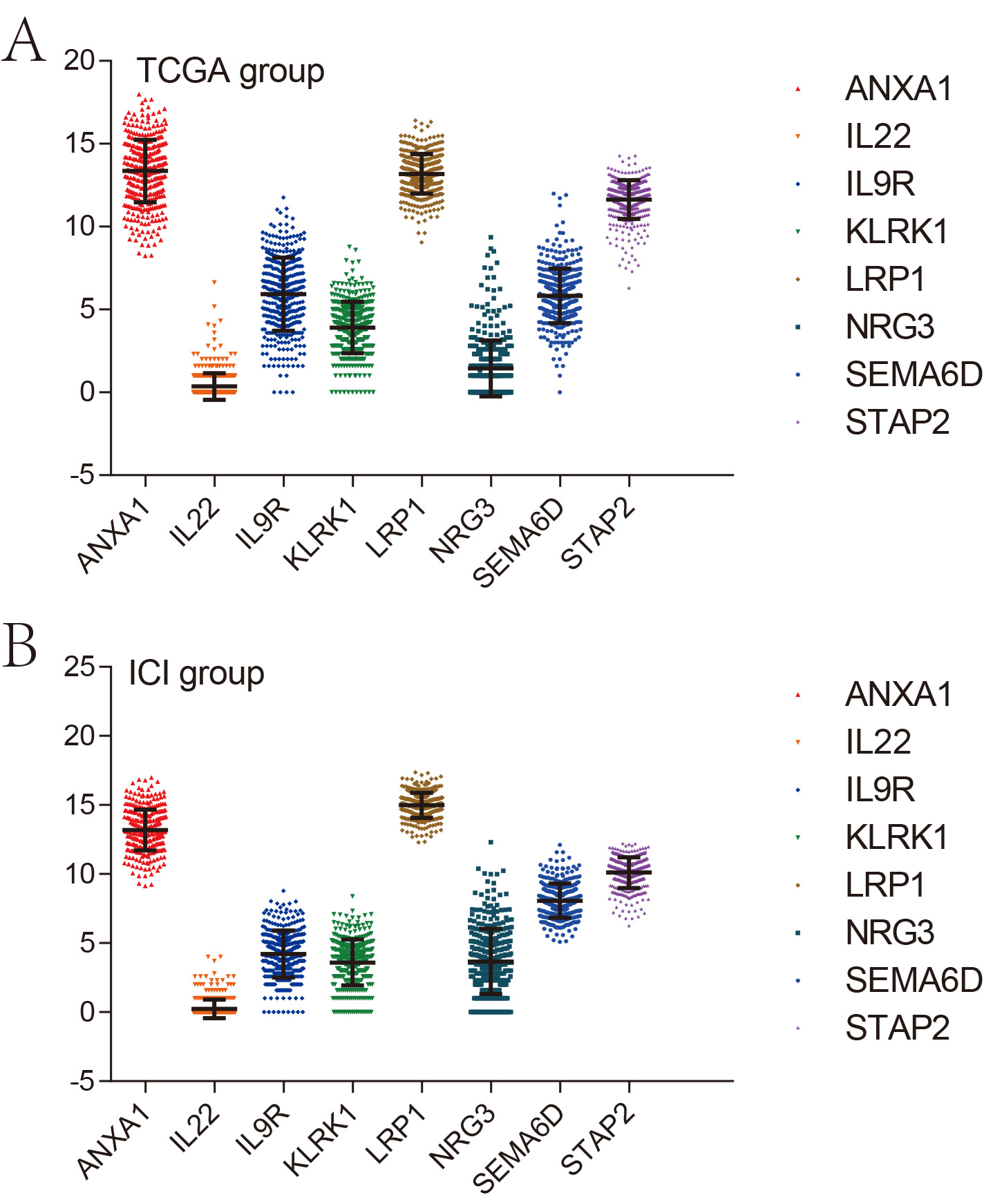

Supplement: Supplementary Figure 1 — Distribution of each DEIG in TCGA set and ICI treatment set. [file Image_1.JPEG]
